# Accuracy of Reported Sizes of the EVO/EVO+ Visian Implantable Collamer® Lenses

**DOI:** 10.7759/cureus.90468

**Published:** 2025-08-19

**Authors:** Matthew Hirabayashi, Gurpal Virdi, Taj Nasser, Peeradol Wattanasirakul, Gregory Parkhurst

**Affiliations:** 1 Ophthalmology, Parkhurst NuVision LASIK Eye Surgery, San Antonio, USA; 2 Ophthalmology, Mason Eye Institute, Columbia, USA; 3 Ophthalmology, Tylock-George Eye Care and Laser Center, Irving, USA; 4 Ophthalmology, University of Missouri School of Medicine, Columbia, USA

**Keywords:** evo visian icl, icl sizing accuracy, implantable collamer lenses (icls), manufacturing precision, measurement discrepancy, toric and spherical icls

## Abstract

Purpose

The purpose of this study is to assess the variability between published manufacturer sizes and actual sizes measured in the operating room (OR) for Implantable Collamer® Lenses (ICLs) (STAAR Surgical, Monrovia, CA).

Methods

A prospective observational study was performed where the diagonal length of 50 ICLs from 50 consecutive cases was measured between June 26, 2023, and November 1, 2023. ICLs were positioned “face down” next to an OR ruler and photographed using the NGENUITY® 3D Visualization System (Alcon, Geneva, Switzerland). These images were processed in Adobe Photoshop 24.6 (Adobe Inc., San Jose, CA) to eliminate perspective distortions, and the images were scaled using known dimensions of the OR ruler. Accurate diagonal measurements were taken using the caliper tool. Measurements were repeated across ICLs in the other direction to ensure consistency. Average size discrepancy, defined as measured ICL size minus published ICL size, was reported along with standard deviation (SD) and confidence interval (CI). A two one-sided t-test (TOST) was used to compare statistical equivalence between spherical and toric ICLs within a 0.017 mm margin.

Results

TOST analysis revealed that the mean measured size of all lens sizes (12.1 mm, 12.6 mm, and 13.2 mm) and types (spherical and toric) is statistically equivalent (α = 0.05) to their labeled size, indicating a high level of manufacturing precision. No ICLs deviated from published measurements by more than 0.05 mm.

Conclusion

The actual sizes of ICLs closely match the published manufacturer sizes, suggesting high manufacturing precision. There is no significant variability between spherical and toric ICLs, supporting the reliability of published sizing for clinical decisions.

## Introduction

Implantable Collamer® Lenses (ICLs) (STAAR Surgical, Monrovia, CA) provide exceptional outcomes for any patient who qualifies for them, despite their typical use in those with high myopia and/or inadequate corneal thickness with contraindications for corneal ablative procedures (e.g., laser-assisted in situ keratomileusis {LASIK}, photorefractive keratectomy {PRK}, and small incision lenticule extraction {SMILE}) [1]. Since their FDA approval in 2005, ICLs have consistently provided excellent postoperative vision with an exceptional safety profile [2]. ICL implantation has been shown by various studies to be effective in correcting low myopia [3,4] and moderate to high myopia [5,6], with more than three million ICLs implanted to date [7]. The newest iteration, the EVO/EVO+ Visian ICLs, has the lowest complication risk thus far and is manufactured in standard sizes measured diagonally across the ICL of 12.1 mm, 12.6 mm, 13.2 mm, and 13.7 mm [8]. Appropriate sizing is critical as this directly influences the vault of the lens, which directly influences the risk of narrowing the angle, contacting the crystalline lens, pigment dispersion, or even endothelial cell loss [9,10]. The International Organization for Standardization (ISO) sets the manufacturing standards for intraocular lenses (IOLs); however, many manufacturers often surpass these established criteria to ensure higher quality and precision [11].

While the explantation/replacement of ICLs due to incorrect size is rare, variability in manufacturing consistency may be a contributing factor that complicates accurate sizing [12]. To our knowledge, ICLs of the same size are expected to have minimal variability, and independent verification can further enhance confidence in clinical decision-making. We sought to measure 50 consecutive ICLs of various sizes to determine the variability between the published manufacturer size and the size measured in the operating room (OR). A sample size of 50 ICLs was selected based on pragmatic feasibility, reflecting a consecutive series of cases over a defined surgical period. While no formal power analysis was performed in advance, this sample was considered adequate to characterize measurement consistency and detect minor deviations between manufacturer-reported and intraoperatively measured ICL dimensions.

## Materials and methods

Study design

A prospective observational study was performed where the diagonal length of 50 ICLs from 50 consecutive cases was measured between June 26, 2023, and November 1, 2023, at a single surgical center.

Ethical consideration

The Advarra Institutional Review Board (Columbia, MD) approved this study (approval number: Pro00073198), and it was conducted in accordance with the tenets of the Declaration of Helsinki and adherent to all Health Insurance Portability and Accountability Act of 1996 (HIPAA) regulations. Informed consent for treatment and open-access publication was obtained or waived from all patients before the operation.

ICL measurement and statistical analysis

**Figure 1:**
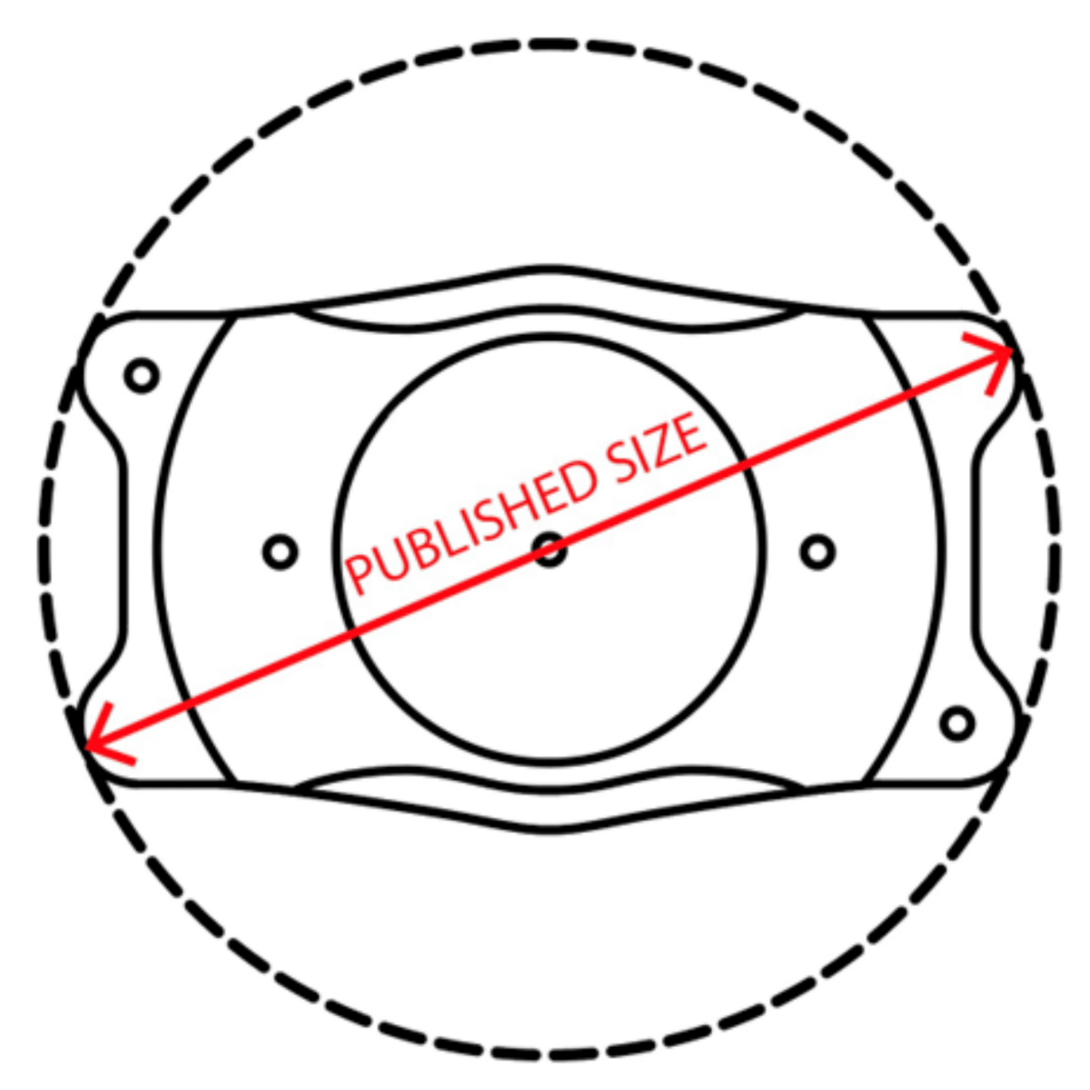
Diagonal measurement representing the reported ICL sizes The photo of ICL viewed under the NGENUITY® 3D Visualization System was measured diagonally using Adobe Photoshop 24.6. The ICL photo was manipulated to minimize perspective distortion. Image scale was adjusted to the known dimensions of the OR ruler. Measurement was repeated across the ICL in the other direction to ensure that equal measurements were obtained. Image credit: Matthew Hirabayashi, MD ICL, Implantable Collamer® Lens; OR, operating room

**Figure 2:**
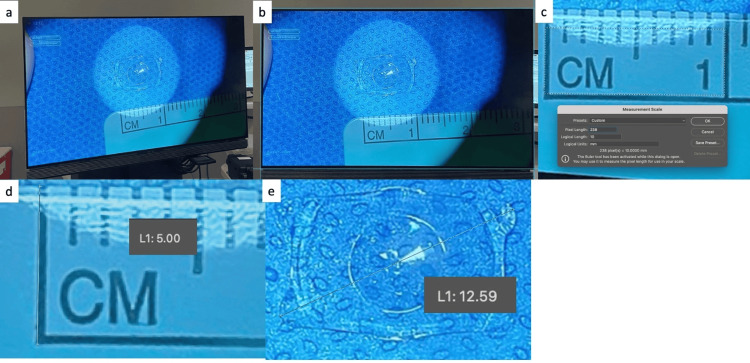
ICL measurement summary Summary of the methodology using Adobe Photoshop to measure ICL size from a photograph taken in the OR immediately preceding implantation. (a) Raw image = initial photograph from the OR. (b) Corrected for perspective = the image is processed to correct for perspective distortions. (c) Setting scale = a scale is set for the image using known measurements (the OR ruler). (d) Scale check = the scale is checked against known lengths (e.g., height of ruler marks) for accuracy. (e) Measuring ICL = the ICL is measured diagonally, bisecting the central port at maximum length with the caliper tool through both sets of corners. Image credit: Matthew Hirabayashi, MD ICL, Implantable Collamer® Lens; OR, operating room

A method to quickly and accurately measure the ICLs was devised to provide minimal disruption to OR flow. Immediately after removing the ICL from its packaging vial and before loading it in the cartridge, the ICL was positioned “face down” on the central port next to an OR ruler. This setup was viewed through the NGENUITY® 3D Visualization System (Alcon, Geneva, Switzerland) on the same monitor used for surgery, and a technician or a nurse photographed the television. The ICL was then implanted in the typical fashion. The photos were imported into Adobe Photoshop 24.6 (Adobe Inc., San Jose, CA) and manipulated according to Figure 1 such that perspective distortions were eliminated. After adjusting the image scale using the known dimensions of the OR ruler, the caliper tool was used to take an accurate measurement of the diagonal length of the ICL. This measurement was repeated across the ICL in the other direction to ensure that equal measurements were obtained. A summary of the methodology is presented in Figure 2.

Average size discrepancy, defined as measured ICL size minus published ICL size, was reported along with standard deviation (SD) and confidence interval (CI). The sizing equivalence within a 0.017 mm margin between spherical and toric ICLs was compared using a two one-sided t-test (TOST). The null hypothesis was defined as the difference between measured and published lens size is greater than or equal to 0.017 mm. The alternate hypothesis was defined as the difference between measured and published lens size is less than 0.017 mm. A p-value of <0.05 was considered significant. All statistics were performed in Microsoft Excel (Microsoft Corp., Redmond, WA).

## Results

This study measured 50 ICLs from consecutive cases with 74% toric ICLs (n = 37), 26% spherical ICLs (n = 13), 18% 12.1 mm ICLs (n = 9), 48% 12.6 mm ICLs (n = 24), 34% 13.2 mm ICLs (n = 17), and no 13.7 mm ICL, as shown in Table 1.

**Table 1:** Characteristics of the measured EVO ICLs ICLs: Implantable Collamer® Lenses

Total ICL, n	50
Toric ICL, n (%)	37 (74%)
Spherical ICL, n (%)	13 (26%)
12.1 mm ICL, n (%)	9 (18%)
12.6 mm ICL, n (%)	24 (48%)
13.2 mm ICL, n (%)	17 (34%)
13.7 mm ICL, n (%)	0 (0%)

The spectrum of options was well represented. For each lens size, the mean measured diameter, standard deviation (SD), and 95% confidence interval (CI) were calculated to assess central tendency, variability, and the precision of measurements and summarized in Table 2. The 12.1 mm ICLs had a mean of 12.10 mm (SD = 0.02), while the 12.6 mm and 13.2 mm lenses had means of 12.61 mm (SD = 0.02) and 13.21 mm (SD = 0.01), respectively. The mean measured lens diameters were closely aligned with their corresponding published values across all ICL sizes. No ICLs of the 50 evaluated deviated from published measurements by more than 0.05 mm. The 95% confidence intervals for all sizes included the published size, indicating that the measured mean closely resembles the corresponding published size.

**Table 2:** Summary of ICL measurements Standard deviations of all lens sizes are within ±0.02 mm; 95% confidence interval of each ICL size contains the corresponding target size ICLs: Implantable Collamer® Lenses

ICL	Mean (mm)	Standard Deviation (mm)	95% Confidence Interval (mm)
12.1 mm ICLs	12.10	±0.02	12.09, 12.11
12.6 mm ICLs	12.61	±0.02	12.60, 12.62
13.2 mm ICLs	13.21	±0.01	13.20, 13.21

Equivalence testing using the TOST procedure was performed with a predefined equivalence margin of ±0.017 mm for all lens sizes (12.1 mm, 12.6 mm, and 13.2 mm) and lens type (spherical and toric). The result of the TOST analysis is summarized in Table 3 and Table 4, with a p-value of <0.05 considered statistically significant. P-values of all groups were under 0.05, ranging from 0.00019 to 0.033, signifying equivalence in all ICL groups. The TOST analysis revealed that the observed deviation within each ICL group is statistically insignificant. Collectively, these findings confirm that the measured lens diameters are both accurate and consistent with their labeled dimensions. The data support the precision and reliability of lens manufacturing across all evaluated groups.

**Table 3:** Size equivalence of all ICL sizes Equivalence testing using the two one-sided t-test (TOST) procedure was performed with a predefined equivalence margin of ±0.017 mm for all lens sizes (12.1 mm, 12.6 mm, and 13.2 mm). P < 0.05 is considered significant ICL: Implantable Collamer® Lens

Published ICL Size (mm)	Mean Difference (mm)	Margin (±mm)	TOST P-value
12.1	0.0033	0.017	0.017
12.6	0.010	0.017	0.024
13.2	0.0071	0.017	0.00062

**Table 4:** Size equivalence of all ICL types Equivalence testing using the two one-sided t-test (TOST) procedure was performed with a predefined equivalence margin of ±0.017 mm for all lens types (spherical and toric). P < 0.05 is considered significant ICL: Implantable Collamer® Lens

ICL Type	Mean Difference (mm)	Margin (±mm)	TOST P-value
Spherical	0.0092	0.017	0.033
Toric	0.0073	0.017	0.00019

## Discussion

ICL is a phakic intraocular lens implanted in the posterior chamber to correct refractive errors [13]. Unlike other refractive surgeries (LASIK, PRK, and SMILE), ICL surgery preserves corneal tissue, allowing for near total reversibility and flexibility for additional surface ablation procedures or premium IOLs in the future, which contributes to its growing popularity [7,13]. With the increasing implantation of ICL, its safety profile and postoperative outcomes have become an important clinical discussion. ICL is implanted inside a “vault,” defined as the distance between the anterior surface of the natural lens and the posterior surface of the ICL [14], with the ideal distance between 250 and 750 µm [15]. Deviation from the ideal vault distance is an important factor in the development of postoperative complications, as a high vault distance can result in angle closure and elevated IOP [16] while a low vault distance can risk contact with the crystalline lens and, in turn, predispose to cataract formation [17,18].

The accurate preoperative prediction of vault size is especially challenging for new surgeons, as achieving optimal vault size requires precise measurements of biometric parameters such as anterior chamber depth [19], white-to-white distance [20], and sulcus-to-sulcus distance [21]. The use of verified ICL sizes can significantly simplify this process by removing one major variability, thereby easing the learning curve for new surgeons. Because appropriate ICL sizing is critical for a successful outcome, many groups across the world are seeking solutions to improve accuracy even further [22]. Since the vault is directly correlated to the actual size of the ICL, the validation of the published ICL sizes can reduce concerns over manufacturer variability.

This study aims to assess the variability between published manufacturer sizes and actual sizes of ICLs. The TOST analysis revealed significant equivalence among all lens sizes and types with p-values ranging from 0.00019 to 0.033 (α = 0.05) within a 0.017 mm (17 µm) margin, indicating high manufacturing precision of ICL across all evaluated groups. Given that the ideal vault size is 250-750 µm, the minimal deviation reported in this study supports the reliability of ICL sizing [15].

To our knowledge, this is the first third-party, non-biased evaluation of actual ICL sizes. Using photo-based digital measuring methods, we found that the actual measured ICL size has small variability and is consistently accurate to the published manufacturer data. None of the lenses in the 50 consecutive cases sampled varied by more than 0.05 mm from the published manufacturer size data. The result in this study gives confidence to surgeons that the manufacturer standards for ICL sizing are high and aid in the selection of the proper ICL size to minimize postoperative complications.

Limitations to this study include a small sample size. Additionally, potential sources of error in the methodology highlight the difficulty in accurately measuring the flexible Collamer® material. Measuring them all laying on the central port provided consistency. Similarly, using photo-based measurements has the potential to introduce error as each pixel is eventually assigned a length, but the repeatability of the measurements, their accuracy to manufacturer values, and the standardized way the data were collected lend credibility to the methodology. Alternative methods for measuring size can strengthen these results.

## Conclusions

EVO/EVO+ Visian ICLs, the newest iteration of Implantable Collamer® Lenses, have the lowest postoperative complication risk thus far. The accurate sizing of ICL is crucial to minimize the risk of complications such as developing pupillary block, anterior cataract development, pigment dispersion, or endothelial cell loss. ICL sizing is an evolving topic, and while explantation rates due to incorrect sizes are low, constant diligence regarding potential sources of error is necessary to further improve accuracy. The TOST analysis of this study reports significant equivalence among reported ICL sizes and types within 0.017 mm with p-values ranging from 0.00019 to 0.033 (α = 0.05), signifying high manufacturing precision. Furthermore, this study indicates that no ICLs of the 50 evaluated deviated from published measurements by more than 0.05 mm, pointing to acceptable manufacturing accuracy. This prospective observational study verified in an unbiased manner that unit-to-unit variability is not likely a significant source of error in ICL sizing.
